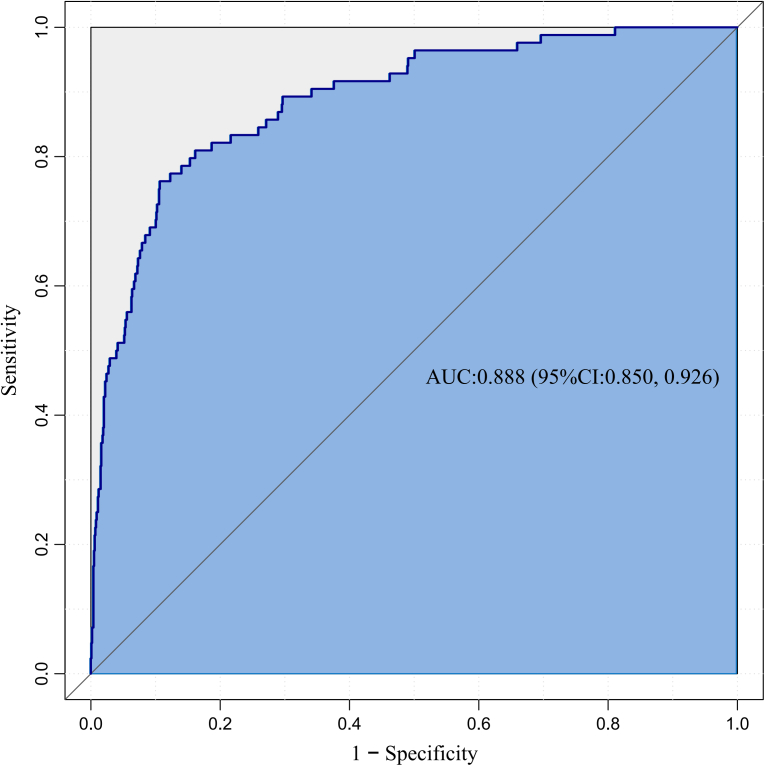# Prior intra-articular sodium hyaluronate injection as a novel predictor of isolated distal deep vein thrombosis after knee surgery: development and validation of a 5-factor nomogram

**DOI:** 10.1016/j.rpth.2026.106619

**Published:** 2026-04-30

**Authors:** Xiaoguang Xie, Jie Lu, Houmei Huang, Yuerong Wang, Menghui Ma, Mengyun Lai, Jing Liu, Xiaojing Zheng

**Affiliations:** 1 Shenzhen Luohu District Hospital of Traditional Chinese Medicine, Shenzhen, China; 2 Gansu University of Traditional Chinese Medicine, Lanzhou, China; 3 Shenzhen Luohu Hospital Group, Luohu People’s Hospital, Shenzhen, China

**Keywords:** hyaluronic acid, knee joint/surgery, nomograms, risk factors, venous thrombosis

## Abstract

**Background:**

Isolated distal deep vein thrombosis (IDDVT) is a frequent but underrecognized complication after knee surgery. Existing prediction tools rarely target distal thrombosis specifically and often omit key perioperative laboratory, inflammatory, and procedural variables.

**Objectives:**

The study’s objective was to pinpoint independent determinants of postoperative IDDVT and to design a personalized predictive nomogram.

**Methods:**

This retrospective analysis enrolled 1068 individuals who received knee operations during the period from 2019 to 2024. Demographic characteristics, laboratory biomarkers, inflammatory indices, anesthesia duration, and a history of intra-articular sodium hyaluronate injection were collected. Least absolute shrinkage and selection operator regression identified the relevant predictors, which were then input into a multivariable logistic regression model to produce the final algorithm. Model performance was evaluated through area under the curve (AUC) analysis, bootstrap-based calibration assessment, decision curve analysis, and examination of clinical impact curve.

**Results:**

IDDVT occurred in 84 patients (7.9%). Five variables were independently associated with IDDVT: D-dimer, fasting plasma glucose, neutrophil-to-lymphocyte ratio, anesthesia duration, and prior intra-articular sodium hyaluronate injection. The nomogram demonstrated excellent discrimination (area under the curve, 0.889; 95% CI, 0.851-0.927) and good calibration (Brier score, 0.052; calibration slope, 1.000). Both decision curve analysis and clinical impact curve demonstrated that the model offered substantial net benefit across threshold probabilities ranging from 1% to 74%.

**Conclusion:**

We developed and validated a 5-factor nomogram for predicting post–knee surgery IDDVT. Prior sodium hyaluronate injection emerged as a significant predictor, likely as a marker of advanced joint pathology. Further prospective studies are needed to integrate this finding into perioperative risk management.

## Essentials

• Five independent predictors of IDDVT after knee surgery were identified.
• Prior intra-articular sodium hyaluronate injection is a novel risk factor.
• A LASSO-based nomogram was developed to predict postoperative IDDVT risk.
• The model showed high accuracy (AUC, 0.889) and strong clinical utility.

## 1 Introduction

When thrombosis occurs only within the infrapopliteal deep venous system, it is classified as isolated distal deep vein thrombosis (IDDVT), a meaningful subgroup of lower-limb deep vein thrombosis (DVT). Although traditionally considered less severe than proximal DVT, distal thrombi may extend proximally or embolize if not promptly recognized and treated [1,2]. Knee procedures—such as total knee arthroplasty, arthroscopy, and ligament repair—markedly elevate venous thromboembolism (VTE) risk owing to perioperative immobility, tourniquet-related venous stasis, and endothelial disruption. Historically, the incidence of postoperative DVT following total knee arthroplasty ranged from 46% to 84% without prophylaxis, and even in modern cohorts using routine thromboprophylaxis, clinically significant VTE events still occur (30-day incidence ≈ 1.19%) [3,4].

Despite increasing awareness of IDDVT, most studies on postoperative DVT have focused on mixed thrombosis populations or proximal events, resulting in limited evidence specific to distal thrombosis after knee surgery. Furthermore, existing prediction models seldom incorporate key perioperative indicators—such as inflammatory biomarkers, coagulation parameters, anesthesia duration, and intra-articular injection history—which restricts their clinical applicability for individualized risk stratification. This knowledge gap contributes to underdiagnosis and may hinder the timely initiation of thromboprophylaxis.

To bridge this gap, we sought to construct a practical predictive model for IDDVT following knee operations. Specifically, we sought to (1) identify perioperative demographic, laboratory, inflammatory, and procedural factors associated with postoperative IDDVT; (2) construct a multivariable logistic regression model based on rigorous variable selection; (3) evaluate model performance in discrimination, calibration, and clinical utility using receiver operating characteristic (ROC) analysis, calibration curves, decision curve analysis (DCA), and clinical impact curves (CIC); and (4) establish an easy-to-use nomogram to support individualized postoperative thrombosis risk estimation.

## 2 Methods

### 2.1 Study design and participants

This retrospective cohort investigation was carried out at the Department of Orthopedics, Shenzhen Luohu District Hospital of Traditional Chinese Medicine, from January 2019 to December 2024. Retrospective data were obtained from adult urban residents who underwent knee surgery. Knee surgery was defined as any operative procedure involving the knee joint performed during the study period. This included knee arthroplasty (total or unicompartmental knee replacement), knee arthroscopy, and other knee procedures such as ligament reconstruction, cartilage repair, or removal of intra-articular foreign bodies. Eligible participants were those aged ≥18 years with complete clinical documentation. Individuals were excluded if they were long-term users of anticoagulants, had a DVT history, or lacked postoperative duplex ultrasonography results. Postoperative prophylactic anticoagulation was not an exclusion criterion. Specifically, patients undergoing total or unicompartmental knee arthroplasty received routine, standardized pharmacologic thromboprophylaxis postoperatively. In contrast, those undergoing arthroscopic or other knee procedures did not receive routine thromboprophylaxis. The outcome of interest was early postoperative IDDVT detected within 7 days after surgery.

**Figure 1:**
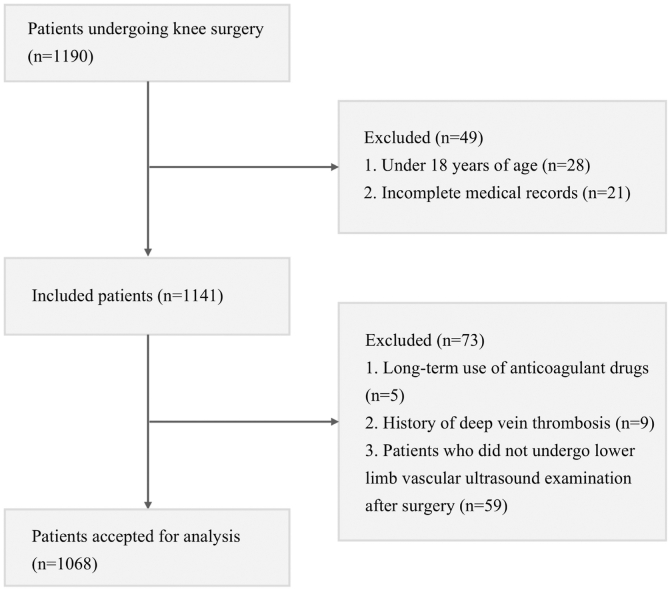
Flowchart of patient selection. Flow diagram illustrating the screening and selection process for patients included in the study. Of 1190 individuals initially assessed, 1068 met the eligibility criteria and were included in the final analysis following exclusions for age <18 years, incomplete medical records, long-term anticoagulant use, prior deep vein thrombosis, or absence of postoperative lower-limb ultrasonography.

Of the 1190 patients reviewed, 49 were excluded due to being under 18 years of age or having incomplete medical documentation. Among the 1141 who remained, another 73 were removed for reasons including prolonged anticoagulant therapy (*n* = 5), established DVT history (*n* = 9), or unavailable postoperative ultrasound findings (*n* = 59). To ensure the absence of pre-existing VTE at baseline and minimize selection bias, all patients underwent routine preoperative lower-extremity compression ultrasonography; those with evidence of pre-existing DVT were strictly excluded from the study. Therefore, 1068 patients comprised the final study cohort. A visual summary of the selection procedure is provided in Figure 1.

This research received approval from the Ethics Committee of Shenzhen Luohu District Hospital of Traditional Chinese Medicine (ID: 2025-LHQZYYYXLL-KY-028) and was carried out in accordance with the principles of the 1975 Declaration of Helsinki. Prior to analysis, all datasets were deidentified by hospital information department personnel to ensure patient privacy. Direct identifiers—including names, national identification numbers, contact details, and medical record numbers—were permanently removed. To further safeguard anonymity, specific calendar dates were converted into relative time intervals (eg, days postsurgery). This anonymization process was completed before data extraction for research purposes, ensuring that the research team remained blind to any identifiable information throughout the study. Consequently, the requirement for informed consent was waived by the Ethics Committee of Shenzhen Luohu District Hospital of Traditional Chinese Medicine, as the study posed no more than minimal risk to participants.

### 2.2 Data collection

Clinical data were sourced from the hospital’s electronic records platform. The dataset comprised demographic information (age, sex, and body mass index [BMI]), first-time laboratory measurements obtained before surgery after admission (D-dimer, fasting plasma glucose [FPG], hemoglobin, platelet count, serum albumin, neutrophil-to-lymphocyte ratio [NLR], and platelet-to-lymphocyte ratio), relevant comorbidities (including hypertension, diabetes, coronary artery disease, hyperlipidemia, hyperuricemia, and lower-limb varicosities), anesthesia duration (in minutes), and medication history such as statins, aspirin, and prior intra-articular sodium hyaluronate administration. A history of intra-articular sodium hyaluronate injection was defined as any documented injection (including viscosupplementation therapy) administered to the affected knee within the 12 months preceding surgery. To ensure data accuracy and minimize recall bias, injection histories were retrieved exclusively from the electronic medical record system—including outpatient procedure logs and pharmacy records—rather than relying on patient self-reports. The primary indications for these injections included symptomatic knee osteoarthritis or recurrent synovitis. All recorded administrations used standard sodium hyaluronate formulations; any off-label medications or alternative intra-articular therapies (eg, corticosteroids or platelet-rich plasma) were strictly excluded from this category.

All postoperative lower-extremity ultrasound examinations were performed by experienced sonographers according to a standardized protocol to screen for DVT. Two investigators independently reviewed all extracted data, and discrepancies were resolved by consensus or by consulting an experienced third reviewer.

### 2.3 Assessment of IDDVT

Per diagnostic guidelines, IDDVT denoted thrombosis restricted to deep calf veins (posterior tibial, peroneal, anterior tibial, gastrocnemius, or soleus) without popliteal or proximal extension and in the absence of pulmonary embolism [[5], [6], [7]]. All patients underwent postoperative lower-limb duplex ultrasonography performed by experienced radiologists within 1 week of surgery, a period during which most postoperative distal thrombi tend to occur [3]. The diagnosis was based on absent venous compressibility, visualization of intraluminal thrombus, or reduced or absent Doppler flow signals. Both symptomatic and asymptomatic IDDVT were included.

At our center, postoperative duplex ultrasonography is routinely performed after knee surgery as part of standardized surveillance for VTE, given that distal DVT is frequently asymptomatic. No proximal DVT events were detected in the cohort. Pulmonary embolism was not observed during hospitalization and was excluded based on clinical evaluation and imaging when clinically indicated. Therefore, the comparison group consisted entirely of patients without postoperative VTE.

### 2.4 Statistical analysis and model building

Continuous measures were analyzed as mean ± SD or as median with IQR depending on distributional characteristics, and statistical comparisons between groups used either the *t*-test or Mann–Whitney U-test. Categorical variables were summarized as frequencies with percentages and examined using chi-square or Fisher exact procedures. To control model complexity, feature selection relied on least absolute shrinkage and selection operator (LASSO) with 10-fold crossvalidation, and the retained predictors were then modeled through multivariable logistic regression. These regression results were subsequently converted into a nomogram that enables clinicians to estimate the likelihood of postoperative IDDVT at the individual level.

Internal validation was used to appraise model performance. Discrimination was determined by the area under the curve (AUC) of the ROC curve with 95% CIs. Calibration was assessed using bootstrap-based calibration plots (500 repetitions). Clinical utility was explored through DCA to quantify net benefit and CIC to compare predicted high-risk counts with observed events.

To evaluate the robustness of the primary predictive model, 2-step sensitivity analyses were conducted. First, a series of clinically significant baseline covariates—including age, sex, BMI, hemoglobin, platelet count, and albumin—were forced into the multivariable model. This was performed to assess the stability of the primary effect estimates and the model’s overall discriminative power. Second, an extended model was developed by further adjusting for postoperative pharmacologic thromboprophylaxis to account for its potential confounding effect on IDDVT risk. Statistical analyses were performed in R software (version 4.4.2; R Foundation for Statistical Computing), and significance was defined as a 2-tailed *P* < .05.

## 3 Results

### 3.1 Baseline characteristics

Of the 1068 patients analyzed, 172 (16.1%) underwent knee arthroplasty (total or partial knee replacement), 773 (72.4%) underwent knee arthroscopy, and 123 (11.5%) underwent other knee procedures, including ligament reconstruction, cartilage repair, and removal of intra-articular foreign bodies. IDDVT occurred in 84 cases, accounting for 7.9%. No cases of proximal DVT or pulmonary embolism were detected during postoperative screening; thus, all thrombotic events identified in this cohort were classified as IDDVT. Clinical symptoms were present in 19 cases (22.6%), but most instances (77.4%, n = 65) were asymptomatic, discovered only via routine postoperative screening. Comparative baseline characteristics for the IDDVT and non-IDDVT cohorts are detailed in Table 1. Compared with patients without IDDVT, those who developed IDDVT were more frequently females (75.0% vs 55.8%; *P* < .001) and were older (58.6 ± 15.5 vs 49.9 ± 16.1 years; *P* < .001).

**Table 1:** Baseline characteristics of patients with and without IDDVT.

Variables	Total	Non-IDDVT^a^	IDDVT	*P*
*N*	1068	984	84	
Sex				<.001
Male	456 (42.7)	435 (44.2)	21 (25)	
Female	612 (57.3)	549 (55.8)	63 (75)	
Age (y)	50.5 ± 16.2	49.9 ± 16.1	58.6 ± 15.5	<.001
BMI (kg/m^2^)	24.5 ± 3.2	24.4 ± 3.2	24.7 ± 2.7	.521
D-dimer (mg/L)	0.6 (0.2–1.3)	0.5 (0.2–1.3)	3.1 (1.1–4.8)	<.001
FPG (mmol/L)	5.4 ± 1.2	5.3 ± 1.0	6.3 ± 2.5	<.001
Hemoglobin (g/L)	133.8 ± 15.7	134.3 ± 15.7	128.4 ± 15.9	<.001
Platelet count	232.9 ± 61.2	232.6 ± 61.7	236.3 ± 55.5	.594
Albumin (g/L)	39.7 ± 3.5	39.9 ± 3.5	38.4 ± 3.7	<.001
NLR	1.8 (1.4–2.4)	1.7 (1.4–2.3)	2.0 (1.5–3.3)	.001
PLR	127.1 ± 53.4	124.7 ± 50.7	154.7 ± 72.9	<.001
Duration of anesthesia	118.8 ± 44.1	114.7 ± 40.5	166.3 ± 55.8	<.001
Previous intra-articular sodium hyaluronate injection				<.001
No	1023 (95.8)	957 (97.3)	66 (78.6)	
Yes	45 ( 4.2)	27 (2.7)	18 (21.4)	
Hypertension				.141
No	603 (56.5)	562 (57.1)	41 (48.8)	
Yes	465 (43.5)	422 (42.9)	43 (51.2)	
Diabetes				.059
No	837 (78.4)	778 (79.1)	59 (70.2)	
Yes	231 (21.6)	206 (20.9)	25 (29.8)	
Coronary heart disease				1
No	1033 (96.7)	951 (96.6)	82 (97.6)	
Yes	35 (3.3)	33 (3.4)	2 (2.4)	
Hyperlipidemia				.485
No	929 (87.0)	858 (87.2)	71 (84.5)	
Yes	139 (13.0)	126 (12.8)	13 (15.5)	
Hyperuricemia				.393
No	718 (67.2)	658 (66.9)	60 (71.4)	
Yes	350 (32.8)	326 (33.1)	24 (28.6)	
Varicose veins of the lower limbs				1
No	1057 (99.0)	973 (98.9)	84 (100)	
Yes	11 ( 1.0)	11 (1.1)	0 (0)	
Statin use				.624
No	1006 (94.2)	928 (94.3)	78 (92.9)	
Yes	62 ( 5.8)	56 (5.7)	6 (7.1)	
Aspirin use				.751
No	1033 (96.7)	952 (96.7)	81 (96.4)	
Yes	35 ( 3.3)	32 (3.3)	3 (3.6)	
Surgical type				
Knee arthroscopy	773 (72.4)	710 (72.2)	63 (75)	.527
Knee arthroplasty	172 (16.1)	162 (16.5)	10 (11.9)	
Other	123 (11.5)	112 (11.4)	11 (13.1)	
Pharmacologic thromboprophylaxis				.275
No	896 (83.9)	822 (83.5)	74 (88.1)	
Yes	172 (16.1)	162 (16.5)	10 (11.9)	

Values are given as *n* (%), mean ± SD, median (IQR).

BMI, body mass index; FPG, fasting plasma glucose; IDDVT, isolated distal deep vein thrombosis; NLR, neutrophil-to-lymphocyte ratio; PLR, platelet-to-lymphocyte ratio.

a The non-IDDVT group consisted of patients without any postoperative venous thromboembolism (no distal deep vein thrombosis [DVT], proximal DVT, or pulmonary embolism).

The IDDVT group exhibited significantly higher preoperative laboratory markers than the non-IDDVT group, including D-dimer (median, 3.1 mg/L [IQR, 1.1–4.8 mg/L] vs median, 0.5 mg/L [IQR, 0.2–1.3 mg/L]; *P* < .001), FPG (6.3 ± 2.5 vs 5.3 ± 1.0 mmol/L; *P* < .001), NLR (median, 2.0 [IQR, 1.5–3.3] vs 1.7 [IQR, 1.4–2.3]; *P* = 0.001), and platelet-to-lymphocyte ratio (154.7 ± 72.9 vs 124.7 ± 50.7; *P* < .001). Conversely, hemoglobin (128.4 ± 15.9 vs 134.3 ± 15.7 g/L; *P* < .001) and albumin levels (38.4 ± 3.7 vs 39.9 ± 3.5 g/L; *P* < .001) were significantly lower in the IDDVT group. Regarding procedural factors, the mean duration of anesthesia was substantially longer among patients with IDDVT (166.3 ± 55.8 vs 114.7 ± 40.5 minutes; *P* < .001).

The 2 groups showed comparable rates of hypertension, diabetes, coronary disease, dyslipidemia, hyperuricemia, lower-extremity varicosities, and aspirin and statin use (all *P* > .05). Prior sodium hyaluronate injections were substantially more prevalent in individuals who developed IDDVT than in those who did not (21.4% vs 2.7%; *P* < .001).

### 3.2 Variable selection

**Figure 2:**
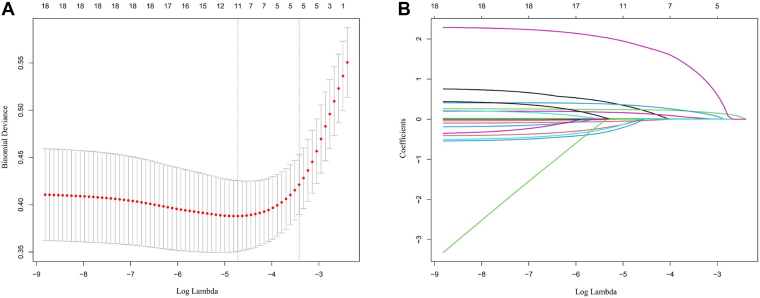
Variable selection using least absolute shrinkage and selection operator (LASSO) regression. (A) Optimal λ value identified by 10-fold crossvalidation. The tuning parameter (λ) was selected based on the minimum binomial deviance. The dotted vertical line represents λ at the minimum deviance, and the second line represents λ under the 1 − SE rule. Five predictors with nonzero coefficients were retained at the optimal penalty value. (B) LASSO coefficient profiles of all candidate variables. Coefficient trajectories for each variable as the penalty increased. As λ increased, the coefficients of less informative variables shrank toward zero. Five variables—D-dimer, fasting plasma glucose, neutrophil-to-lymphocyte ratio, duration of anesthesia, and prior intra-articular sodium hyaluronate injection—remained with nonzero coefficients and were selected for the final model.

To identify the most informative predictors of IDDVT, using the LASSO approach, we first reduced the full set of candidate variables. The penalty parameter *λ* was selected via 10-fold crossvalidation, which indicated its optimal point near log(*λ*) ≈ −5.0 (Figure 2A). According to the 1 − SE selection principle, 5 predictors with coefficients that did not shrink to zero were retained.

As shown in the coefficient profile plot (Figure 2B), coefficients of less relevant variables shrank toward zero as the penalty increased. The 5 predictors retained for model construction were duration of anesthesia, NLR, FPG, D-dimer, and prior intra-articular sodium hyaluronate injection.

### 3.3 Model development

After LASSO screening, the selected 5 factors were assessed within a multivariable logistic regression framework. Since every factor demonstrated independent significance, all were carried forward into the completed prediction model. Detailed coefficients and effect estimates are shown in Table 2. Higher levels of D-dimer (odds ratio [OR], 1.34; 95% CI, 1.21-1.48), FPG (OR, 1.51; 95% CI, 1.30-1.74), NLR (OR, 1.23; 95% CI, 1.10-1.35), and longer duration of anesthesia (OR, 1.02; 95% CI, 1.01-1.03) were independently associated with increased odds of IDDVT. A history of intra-articular sodium hyaluronate injection showed the strongest association (OR, 10.24; 95% CI, 4.65-22.57; *P* < .001).

**Table 2:** Multivariable logistic regression analysis of risk factors associated with IDDVT.

Variable	β (SE)	OR (95% CI)	*Z*	*P*
D-dimer (mg/L)	0.293 (0.051)	1.34 (1.21-1.48)	5.76	<.001
FPG (mmol/L)	0.414 (0.079)	1.51 (1.30-1.74)	5.22	<.001
NLR	0.209 (0.057)	1.23 (1.10-1.35)	3.68	<.001
Duration of anesthesia (min)	0.017 (0.003)	1.02 (1.01-1.03)	5.97	<.001
Prior intra-articular sodium hyaluronate injection (yes vs no)	2.326 (0.401)	10.24 (4.65-22.57)	5.81	<.001

FPG, fasting plasma glucose; IDDVT, isolated distal deep vein thrombosis; NLR, neutrophil-to-lymphocyte ratio; OR, odds ratio.

**Figure 3:**
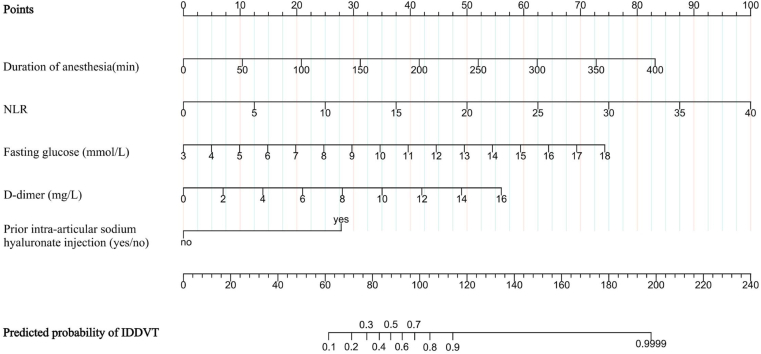
Nomogram for predicting postoperative IDDVT. The nomogram integrates 5 independent predictors: D-dimer, fasting plasma glucose, NLR, duration of anesthesia, and prior intra-articular sodium hyaluronate injection. To use the nomogram, locate the value of each predictor on its respective axis and draw a vertical line upward to the “Points” scale. The sum of these individual scores is then projected onto the “Total points” axis to determine the corresponding probability of IDDVT. Higher total scores indicate an increased risk of postoperative thrombosis. IDDVT, isolated distal deep vein thrombosis; NLR, neutrophil-to-lymphocyte ratio.

We generated a 5-factor nomogram to facilitate individual prediction of postoperative IDDVT (Figure 3). Each component is weighted according to its regression coefficient, and the cumulative score is translated into a corresponding risk estimate. The final logistic regression formula was as follows:

logit(P)=−8.58+0.293×D-dimer+0.414×FPG+0.209×NLR+0.017×durationofanesthesia+2.326×sodiumhyaluronateinjection(yes=1).

$$P=\frac{e^{\text{logit}\left(P\right)}}{1+e^{\text{logit}\left(P\right)}}\text{.}$$

For example, a patient with a D-dimer of 2.0 mg/L, FPG of 6.0 mmol/L, NLR of 3.0, anesthesia duration of 120 minutes, and prior sodium hyaluronate injection had a calculated logit(*P*) of −0.517, corresponding to a predicted IDDVT probability of 37.4%.

### 3.4 Model performance evaluation

#### 3.4.1 Discrimination

**Figure 4:**
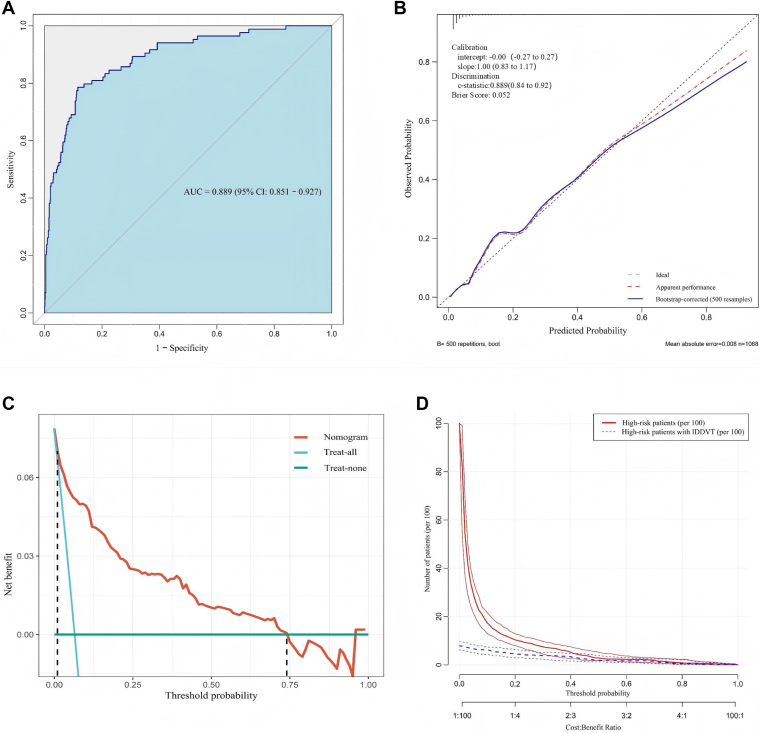
Performance of the IDDVT prediction model. (A) The receiver-operating characteristic curve demonstrates excellent discrimination, with an AUC of 0.889 (95% CI, 0.851-0.927). At the optimal cutoff probability of 0.10, the model achieved a sensitivity of 78.6% and a specificity of 88.4%. (B) The calibration plot compares predicted probabilities with observed outcomes based on 500 bootstrap resamples. Both the logistic and nonparametric curves closely align with the ideal 45° reference line, indicating high predictive accuracy. Calibration metrics: intercept, 0.000; slope, 1.000; Brier score, 0.052. (C) Decision curve analysis demonstrates that the prediction model provides a higher net benefit than both treat-all and treat-none strategies across a threshold probability range of 1% to 74%, highlighting its clinical utility. (D) The clinical impact curve illustrates the relationship between the number of patients classified as high risk and the actual number of true-positive IDDVT cases across various threshold probabilities. The close alignment between the predicted and actual outcomes indicates reliable risk stratification and high clinical applicability. AUC, area under the curve; IDDVT, isolated distal deep vein thrombosis.

The model demonstrated excellent discriminative performance. The ROC curve (Figure 4A) yielded an AUC of 0.889 (95% CI, 0.851-0.927). At the optimal cutoff value of 0.10, the sensitivity was 78.6% (specifically, 88.4%); positive predictive value, 36.7%; and negative predictive value, 98.0%, resulting in a Youden index of 0.67.

#### 3.4.2 Calibration

Figure 4B shows that the estimated probabilities corresponded well with the observed frequencies of IDDVT. Both the model-based calibration line and the nonparametric trend line remained near the perfect calibration slope, suggesting minimal deviation. The calibration intercept (0.000) and slope (1.000) indicated the absence of systematic overestimation or underestimation. Additional metrics supported good calibration as follows:

• Brier score = 0.052
• Dxy = 0.778 (C-index = 0.889)
• Nagelkerke *R*^2^ = 0.393

#### 3.4.3 Clinical utility

DCA revealed that, across threshold probabilities ranging from 1% to 74%, the model consistently outperformed both universal-treatment and no-treatment strategies in terms of net clinical benefit (Figure 4C). The CIC (Figure 4D) further confirmed that the predicted number of high-risk patients closely matched the observed number of IDDVT cases, supporting the model’s clinical applicability.

### 3.5 Sensitivity analysis

**Supplementary Figure S1:**
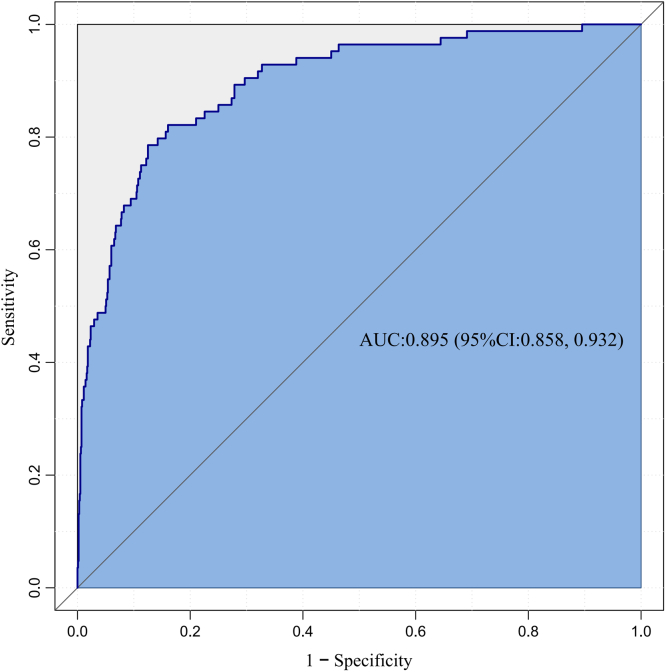


To evaluate the robustness of the predictive model, we performed 2 sensitivity analyses. First, we incorporated clinically significant baseline characteristics—including age, sex, BMI, hemoglobin, platelet count, and serum albumin—into the multivariable model as covariates. The 5 primary predictors (D-dimer, FPG, NLR, duration of anesthesia, and prior sodium hyaluronate injections) remained independently associated with postoperative IDDVT, with effect estimates largely unchanged (Supplementary Table). The model’s discriminative power remained robust (AUC, 0.895; 95% CI, 0.858-0.932), comparable with the primary parsimonious model (Supplementary Figure 1). Second, to account for potential confounding from postoperative thromboprophylaxis, we further adjusted for prophylactic anticoagulation (yes/no). The resulting AUC (0.888; 95% CI, 0.850-0.926) was consistent with the original performance (Supplementary Figure 2). These findings confirm that the nomogram’s predictive value is stable and independent of baseline demographic, hematologic, or interventional variables.

## 5 Discussion

In this retrospective analysis of 1068 patients who undergwent knee surgery, we identified 5 independent predictors of postoperative IDDVT: D-dimer, FPG, NLR, duration of anesthesia, and prior intra-articular sodium hyaluronate injection. When evaluated through DCA, the nomogram built from these variables demonstrated strong discriminative capability, reliable calibration, and substantial clinical relevance. These findings highlight the value of combining perioperative laboratory markers, inflammatory indices, procedural characteristics, and an injection history to improve risk stratification for postoperative IDDVT.

It should be acknowledged that both symptomatic and asymptomatic IDDVT events were included in this study. Although the clinical relevance of asymptomatic distal DVT remains debated, accumulating evidence suggests that a proportion of asymptomatic IDDVTs may progress proximally, recur, or contribute to long-term venous complications if left undetected. In the postoperative setting, early identification of high-risk patients may therefore have clinical value by informing surveillance intensity and individualized thromboprophylaxis strategies rather than immediate therapeutic intervention.

D-dimer, a fibrin degradation product, rises in response to coagulation activation and subsequent fibrinolysis. Our findings regarding D-dimer and FPG align closely with previous literature on postoperative VTE. Postoperative studies in knee surgery populations have consistently reported that patients with DVT exhibit elevated D-dimer levels, which arise from activation of the body’s clot formation and breakdown pathways [8]. Notably, the median preoperative D-dimer level in our IDDVT group was 3.1 mg/L, which might typically raise suspicion of pre-existing thrombosis. However, it is imperative to clarify that all participants in this study underwent mandatory Doppler ultrasound of the lower extremities prior to surgery, and those with any evidence of pre-existing DVT were strictly excluded. This diagnostic protocol ensures that the IDDVT cases identified in our results were indeed new-onset events rather than pre-existing conditions. In this specific surgical population, the elevated preoperative D-dimer likely reflects a systemic prothrombotic and inflammatory primer state—possibly linked to advanced joint pathology or chronic inflammation—which increases vulnerability to acute thrombosis following the additional stress of surgical trauma. Although increased D-dimer levels may occur in various physiological or pathologic conditions, such as aging, renal dysfunction, trauma, or systemic inflammation, low D-dimer levels have a high negative predictive value and allow preliminary exclusion of IDDVT, reducing unnecessary imaging [[9], [10], [11]]. Persistently positive D-dimer after an initial thrombotic episode has been shown to predict recurrence, highlighting its value as a marker of ongoing thrombotic risk [12,13].

Hyperglycemia has similarly been associated with increased VTE risk in orthopedic populations, likely due to metabolic dysregulation, endothelial dysfunction, and platelet hyperreactivity [14]. Patients with impaired glucose regulation or diabetes often demonstrate a markedly prothrombotic milieu characterized by heightened platelet reactivity, increased expression of procoagulant markers, and impaired fibrinolysis. These changes, largely mediated by insulin resistance and chronic inflammation, directly modify coagulation factor activity and clot structure, thereby facilitating venous thrombosis [15,16].

The identification of NLR as a significant predictor echoes prior research associating elevated NLR with postoperative DVT [8], further underscoring the key contribution of inflammatory processes to thrombus formation. A high NLR reflects systemic inflammatory activation that can impair endothelial integrity, promote neutrophil-mediated injury, and increase prothrombotic microparticles, jointly enhancing thrombotic susceptibility [17].

Prolonged anesthesia duration also emerged as an important predictor, consistent with earlier studies demonstrating that extended operative or anesthesia time enhances venous stasis and endothelial injury, thereby promoting thrombus formation [4]. Virchow triad remains a foundational framework for understanding thrombogenesis. Prolonged anesthesia contributes to extended immobilization, reduced venous return, and intraoperative hypoperfusion—mechanisms that collectively promote venous stasis and increase the risk of IDDVT. This association has been widely documented across multiple surgical populations [[18], [19], [20]].

Perhaps, the most notable finding of this study is the identification of prior intra-articular sodium hyaluronate injection as a strong independent predictor of postoperative IDDVT. Evidence linking Visco supplementation to venous thrombosis is extremely limited, consisting mainly of isolated case reports describing DVT following knee hyaluronate injection and pulmonary embolic events after therapeutic sodium hyaluronate administration [21,22]. In addition, postmarketing safety surveillance systems have reported sporadic cases of extensive leg thrombosis after hyaluronate injections [23]. Although rare, these events suggest that thrombotic complications of Visco supplementation may be underrecognized.

The mechanism underlying this association is unclear. Rather than indicating a direct pharmacologic effect, sodium hyaluronate injection may serve as a surrogate marker for more advanced osteoarthritis or recurrent synovitis. These conditions are characterized by chronic inflammation, synovial hypertrophy, altered intra-articular pressure, and microvascular disturbances, all of which can impair venous drainage and promote local stasis in the calf venous system. Such pathophysiological changes may predispose to postoperative distal thrombosis. While causality cannot be inferred, this previously unreported clinical association warrants prospective validation and may represent an overlooked element of preoperative thrombotic risk assessment.

The proposed nomogram may serve as a practical tool for postoperative risk stratification of IDDVT after knee surgery. By identifying patients at higher predicted risk, the model could help inform the intensity of surveillance and guide individualized consideration of preventive strategies. However, clinical implementation should be approached cautiously, as external validation and prospective impact studies are required to confirm its generalizability and whether risk-guided management improves patient outcomes.

Although postoperative pharmacologic thromboprophylaxis differed by surgical procedure in this cohort, sensitivity analysis incorporating thromboprophylaxis as a covariate demonstrated similar model performance. This suggests that the identified predictors capture postoperative IDDVT risk largely independently of procedure-specific prophylaxis strategies. A few limitations merit attention. First, the single-center, retrospective design restricts the broader generalizability of these results. Second, we were unable to further stratify injection exposure into shorter time windows (eg, within 6 months) because the exact timing of prior injections was not consistently available in all medical records. Future prospective studies with standardized exposure documentation are warranted. Third, the absence of an external validation cohort restricts the ability to confirm the nomogram’s broader applicability. Fourth, ultrasound screening is performed within the first week after surgery; therefore, delayed DVT events occurring after discharge may be missed, leading to an underestimation of the true incidence rate. Fifth, our hospital routinely performs preoperative lower-extremity ultrasound examinations to rule out preoperative thrombosis; however, this practice is not universally adopted by all hospitals. Therefore, our findings may have limited applicability in regions where preoperative ultrasound screening is not routinely performed. Finally, this study focused solely on IDDVT and did not examine progression to proximal DVT or pulmonary embolism, potentially underestimating clinical impact.

Future prospective, multicenter studies are required to validate the nomogram and evaluate whether risk-stratified thromboprophylaxis based on model predictions improves postoperative outcomes. Research exploring whether the identified predictors, particularly sodium hyaluronate injection, also predict proximal extension or symptomatic pulmonary embolism would further enhance clinical relevance. Integration of intraoperative hemodynamic monitoring, mobility tracking, and emerging biomarkers may further improve predictive accuracy.

## 6 Conclusion

In conclusion, we developed a 5-factor nomogram that accurately predicts postoperative IDDVT after knee surgery. Notably, prior intra-articular sodium hyaluronate injection emerged as a strong predictor, which may reflect more advanced joint degeneration or chronic synovitis rather than a direct causal effect. This novel association warrants further prospective investigation and may help refine perioperative risk stratification.

## Figures and Tables

**Supplementary Figure S2:**