# Supplementary material for: Prior intra-articular sodium hyaluronate injection as a novel predictor of isolated distal deep vein thrombosis after knee surgery: development and validation of a 5-factor nomogram
Source: Res Pract Thromb Haemost. 2026 Apr 30;10(4):106619. doi: 10.1016/j.rpth.2026.106619 (PMC13224345; doi:10.1016/j.rpth.2026.106619)
Supplement: Supplementary Table and Figures [file mmc1.docx]

# **Appendix 1: Supplementary Tables**

Supplementary Table S1. Sensitivity analysis including clinically important covariates.

| **Variables** |  |  | **OR (95%CI)** |  | **OR (95%CI)** |  |
| --- | --- | --- | --- | --- | --- | --- |
|  | **No** | **n.event_%** | **Crude** | ***P*-value** | **Adjust** | ***P*-value** |
| **D-dimer (mg/L)** | 1068 | 84 (7.9) | 1.48 (1.35~1.61) | <0.001 | 1.43 (1.3~1.58) | <0.001 |
| **FPG (mmol/L)** | 1068 | 84 (7.9) | 1.47 (1.29~1.67) | <0.001 | 1.48 (1.28~1.71) | <0.001 |
| **NLR** | 1068 | 84 (7.9) | 1.26 (1.15~1.38) | <0.001 | 1.26 (1.14~1.39) | <0.001 |
| **Duration of anesthesia (min)** | 1068 | 84 (7.9) | 1.02 (1.02~1.03) | <0.001 | 1.02 (1.02~1.03) | <0.001 |
| **Previous intra-articular sodium hyaluronate injection** |  |  |  |  |  |  |
| No | 1023 | 66 (6.5) | 1(Ref) |  | 1(Ref) |  |
| Yes | 45 | 18 (40) | 9.67 (5.06~18.45) | <0.001 | 7.04 (3.6~13.74) | <0.001 |

Multivariable logistic regression results from the sensitivity model incorporating clinically important baseline variables (age, sex, BMI, hemoglobin, platelet count, and albumin) in addition to the five predictors identified by LASSO selection. The five core predictors (D-dimer, fasting plasma glucose, NLR, anesthesia duration, and prior intra-articular sodium hyaluronate injection) remained independently associated with postoperative IDDVT, with effect estimates consistent with the primary model.

Abbreviations: NLR, neutrophil-to-lymphocyte ratio; FPG, fasting plasma glucose; IDDVT, isolated distal deep vein thrombosis; OR, odds ratio; CI, confidence interval; Ref, reference category; LASSO, least absolute shrinkage and selection operator; BMI, body mass index.

# **Appendix 2: Supplementary Figures**

**Supplementary Figure S1.** **ROC curve of the sensitivity model including clinical covariates**
Receiver operating characteristic (ROC) curve of the extended sensitivity model. This model incorporates baseline clinical variables—including age, sex, BMI, hemoglobin, platelet count, and serum albumin—in addition to the five primary predictors from the original nomogram. The model demonstrated robust discriminatory power with an AUC of 0.895 (95% CI: 0.858–0.932), confirming the stability of the prediction model after adjusting for key baseline characteristics.

Abbreviations: AUC, area under the curve; BMI, body mass index; CI, confidence interval; ROC, receiver operating characteristic.

**Supplementary Figure S2**. **ROC curve of the sensitivity analysis model including pharmacologic thromboprophylaxis**

The Receiver Operating Characteristic (ROC) curve illustrates the model's performance after incorporating postoperative pharmacological prophylaxis (yes/no) as a potential confounder. The area under the curve (AUC) is 0.888 (95% CI: 0.850–0.926), demonstrating that the model’s discriminatory power remains robust and is not materially affected by postoperative anticoagulation therapy. Abbreviations: AUC, area under the curve; CI, confidence interval; ROC, receiver operating characteristic.
